# Uncertainty Quantification in Simulations of Epidemics Using Polynomial Chaos

**DOI:** 10.1155/2012/742086

**Published:** 2012-08-15

**Authors:** F. Santonja, B. Chen-Charpentier

**Affiliations:** ^1^Department of Statistics and Operational Research, University of Valencia, Dr. Moliner 50, 46100 Burjassot, Valencia, Spain; ^2^Department of Mathematics, University of Texas at Arlington, Arlington, TX 76019-0408, USA

## Abstract

Mathematical models based on ordinary differential equations are a useful tool to study the processes involved in epidemiology. Many models consider that the parameters are deterministic variables. But in practice, the transmission parameters present large variability and it is not possible to determine them exactly, and it is necessary to introduce randomness. In this paper, we present an application of the polynomial chaos approach to epidemiological mathematical models based on ordinary differential equations with random coefficients. Taking into account the variability of the transmission parameters of the model, this approach allows us to obtain an auxiliary system of differential equations, which is then integrated numerically to obtain the first-and the second-order moments of the output stochastic processes. A sensitivity analysis based on the polynomial chaos approach is also performed to determine which parameters have the greatest influence on the results. As an example, we will apply the approach to an obesity epidemic model.

## 1. Introduction

Epidemiological mathematical models based on ordinary differential equations are usually used to understand the processes involved in the transmission of diseases [1]. The coefficients of these equations have traditionally been considered deterministic, that is, they have been assumed to be known and have no variation; see, for example, [2, 3]. However, in many situations, equations with random coefficients are better suited in describing the real behavior of quantities of interest than their counterparts with deterministic coefficients. Therefore, considering randomness is particularly important. A probabilistic description provides a more natural and realistic portrayal. Additionally, in the case of multiple uncertain parameters, a probabilistic approach is necessary to avoid unreasonable conservatism.

Differential equations where some or all of the coefficients are considered random variables or that incorporate stochastic effects (usually in the form of white noise) have been increasingly used in the last few decades to deal with errors and uncertainty see [4, 5].

Monte Carlo methods [6, 7] have been used for many years to perform simulations when random effects were involved. They are simple to implement and understand but require many realizations due to their slow convergence rate and hence tend to be expensive. Other methods that have been developed and used are, for example, moment methods [8, 9] and polynomial chaos methods see [10, 11] and the references therein. Moment methods approximations use Taylor series expansions about the mean value of the input parameters. The first-order moment is the deterministic value of the output parameter obtained at the mean of the input, while evaluation of the higher order moments requires computation of sensitivities. The drawback of this approach, is that it is intrinsically limited to small perturbations; it also becomes complicated beyond second-order expansions [4, 12]. In the polynomial chaos approach a high-order representation is far easier to construct—the equations are basically the same at any order the difference lies only in the number of terms to be considered and are of the same form as the corresponding deterministic equations. So there is no need to develop new algorithms and numerical methods. High-order moments are easily accessible, and the spectral convergence of the stochastic approximation guarantees that of high accuracy can be obtained even with a small number of terms see [12, 13] for computational results. An alternative approach is to add white noise terms and thus obtain a system of stochastic differential equations, see, for example, [14, 15] for applications to epidemic models. For discrete population, models can also incorporate randomness. For example, micromodels [16, 17] can be used to model interactions between individuals given by random parameters.

In this paper, we will use the polynomial chaos approach to study this type of epidemiological models with randomness, due to its simplicity. The computational cost can be high if many random parameters are considered, and high-order expansions are used. But in our problem this was not the case. The polynomial chaos method applied to a system of ordinary differential equations with random equations is based on expanding the random coefficients and the unknown variables in terms of orthogonal polynomials of random variables. For example, if a random coefficient has a normal distribution, the Hermite polynomials should be used since they form an orthogonal basis with the normal distribution as the weight. These expansions are then substituted into the differential equations, and the orthogonality is used to obtain a system of the differential equations of the same form as the deterministic model for the unknown coefficients of the expansions. These equations can then be solved using the same numerical methods used for the deterministic case. More details are given in Section 3. As an example, we analyze the time evolution of a system of ordinary differential equations with random transmission parameters designed to understand an obesity epidemic. A deterministic version of the obesity mathematical model considered was presented in [18].

This polynomial chaos technique allows us to consider that the transmission parameters in an epidemiological model are random variables and obtain the evolution of the epidemic and its predictions considering the effects of these randomness. Additionally, the quantification of the effects of the random transmission parameters on the variance of the response of the epidemiological model can also be analyzed calculating the polynomial chaos-based Sobol's indices. These indices are based on the decomposition of the variance of the output as a sum of contributions of each input variable. Taking into account this decomposition, Sobol's indices allow us to quantify the rate defined by the variance related to each parameter and the total variance of the output.

Therefore, this approach is useful to predict the evolution of an epidemic considering the effects of the randomness and to quantify the effects of the random transmission parameters on epidemic evolution (sensitivity analysis).

This paper is structured as follows. In Section 2, a test mathematical model for obesity epidemic is briefly described. The polynomial chaos approach is presented in Section 3.  Section 4 is devoted to numerical results. Finally, conclusions are considered.

## 2. Epidemiological Models

 Classical models of disease dynamics rely on systems of differential equations that divide the number of individuals in various categories through continuous variables allowing for infinitesimal population densities. The origin of these models is commonly traced back to the well-known pioneer work of Hethcote [1]. In this work, they obtained the epidemic threshold result that the density of susceptible population must exceed a critical value in order for an epidemic outbreak to occur.

Some of the assumptions in this type of models are: (i) The number of individuals grows without bound in a Malthusian way; this is modeled by a linear term. (ii) The effect of the disease (the transit to *infected* population) is modeled by a nonlinear term proportional to the *infected* and *noninfected* populations. (iii) The death rate results in exponential decay and is modeled by a linear term.

### 2.1. Obesity Model

 The population with excess weight is growing at a worrying rate in developed and developing countries [20]. The obesity epidemic is becoming a serious health concern not only from the individual health point of view but also from the public socioeconomic one, and it is considered that the study of obesity is of the highest priority, evaluating its magnitude and proposing effective strategies in order to invert this trend in the next few years.

The obesity model used to present the possibilities of the polynomial chaos approach was proposed in [18] to understand the dynamics of the obesity epidemic. This model was defined for individuals aged 24–65 years old. They are divided into three subpopulations using their Body Mass Index size (BMI = weight/height^2^), where the weight is in kilograms and the height in meters: *N*: individuals with normal weight (BMI < 25): *S*, overweight people (25 ≤ BMI < 30), and *O* obese individuals (BMI ≥ 30). The transitions between these different subpopulations are described by the following system of differential equations (*t*, time in weeks):

(1) $$\begin{array}{c} N^{\prime }\left(t\right)=\mu N^{0}-\mu N\left(t\right)-\beta N\left(t\right)\left[S\left(t\right)+O\left(t\right)\right]+\rho S\left(t\right),\\ S^{\prime }\left(t\right)=\mu S^{0}+\beta N\left(t\right)\left[S\left(t\right)+O\left(t\right)\right]-\left[\mu +\gamma +\rho \right]S\left(t\right)+\epsilon O\left(t\right),\\ O^{\prime }\left(t\right)=\mu O^{0}+\gamma S\left(t\right)-\left[\mu +\epsilon \right]O\left(t\right). \end{array}$$

The time invariant parameters of this system of equations are as follows.

- *ϵ*: rate at which an obese adult with healthy lifestyle becomes a overweight individual.
- *μ*: average stay time in the system of 24–65 years old adults.
- *ρ*: rate at which a overweight individual moves to the normal weight sub-population.
- *β*: transmission rate due to social pressure to adopt an unhealthy lifestyle (TV, friends, family, job, etc.).
- *γ*: rate at which an overweight 24–65 years old adult becomes an obese individual by unhealthy lifestyle.
- *N* ^0^: proportion of normal-weight individuals coming from the 23-year-old age group.
- *S* ^0^: proportion of overweight individuals coming from the 23-year-old age group.
- *O* ^0^: proportion of obese individuals coming from the 23-year-old age group.

The values of these parameters for the region of Valencia (Spain) were determined by health survey for the region of Valencia, Spain, year 2000 and year 2005 [19] and a technical report published by Arrizabalaga et al. [21]. To be precise, we take into account the weekly growth of the average weight of a 24–65-year-old adult in the region of Valencia, and the mean time that an individual takes after he/she stops physical activity to start again. Additionally, we consider that an overweight individual takes 24 weeks to transit from obese to overweight subpopulation by physical activity and healthy nutritional habits. We show them in Table 1. For more details about the parameter estimations see [18].

 Parameters, *μ*, *γ*, *ϵ*, and *ρ* can be interpreted as the mean length of the transit period between two subpopulations (weeks^−1^). Note that length of the transit period for a subpopulation is usually assumed to follow an exponential distribution [22].

The initial conditions of the system are also defined by health survey for the region of Valencia, Spain, year 2000. In this case,

(2) $$\begin{array}{c} N\left(t=0\right)=0.522,\;\;S\left(t=0\right)=0.362,\\ O\left(t=0\right)=0.116, \end{array}$$

that is, in the region of Valencia, 52.2% was normal-weight population, 36.2% was overweight population, and 11.6% was obese population in year 2000.

Note that taking into account the differential equations of the model (1), the parameter values (Table 1), and the initial conditions shown above, we can predict obesity incidence in the next few years.

## 3. Random Transmission Parameters and Polynomial Chaos

 It is necessary to introduce randomness in the model (1) since the parameters involved have some degree of uncertainty due to sampling, rounding, and other errors. We consider the transmission parameters of the model (*β*, *γ*, *ϵ*, and *ρ*) as random variables with a certain probability distribution. The proportions of individuals coming from the 23-year-old group (*N* ^0^, *S* ^0^, *O* ^0^) will not be considered random since they can be determined with much more accuracy than the aforementioned parameters. The equations also require initial values of the three sub-populations *N*(*t* = 0), *S*(*t* = 0), and *O*(*t* = 0). These values can also be determined with more accuracy than the transmission parameters and will, therefore, not to be considered random. In both cases (proportions of individuals coming from the 23-year-old group and initial values of the model), the values are estimated considering a representative sample of Valencian population with a sample size of 4,319 individuals. In addition, *N* ^0^, *S* ^0^, *O* ^0^, *N*(0), *S*(0), and *O*(0) are determined by the given population that we are studying, and we are interested in investigating the effects of changes in the transmission parameters on the future values of the three sub-populations. So by only considering that the transmission parameters are random, we take into account the largest sources of uncertainty, while keeping the model relatively simple.

In many situations, the number of data points available is very small, so it is not possible to establish the type of distributions satisfied by the random parameters. This is also true in our case where the number of data points for the transmission parameters is so scarce that is not possible to have a well-defined type of distribution. In this work, we only have the information of one value to estimate the probabilistic distribution of the parameters, the values shown in Table 1. We have not a lot of information to do it. Therefore, we consider a noninformative distribution, the uniform probability distribution. As it is commented in [23, 24], this is a habitual consideration to estimate the parameters of a mathematical model when we have not a lot of information.  Table 2 shows details about the assumed probability distribution of the transmission parameters. Note that for each parameter *θ*, with values *β*, *γ*, *ϵ*, and *ρ*, the maximum likelihood estimation of Uniform (0, *θ*) is the maximum of the sample considered, that is, the only value of the sample is the known value of the parameter. In this case, the expected value of each parameters is a half of its known value. Therefore, if we consider the distribution defined by Uniform (0, 2*θ), we have that its expected value is the known value of the parameter.

Therefore, we consider that the transmission parameters of the model *β*, *γ*, *ϵ*, and *ρ* are random variables depending on the outcome *w* of an experiment, *β* = *β*(*w*), *γ* = *γ*(*w*), *ϵ* = *ϵ*(*w*), and *ρ* = *ρ*(*w*), and the populations *N*(*t*; *w*), *S*(*t*; *w*), and *O*(*t*; *w*) then become stochastic processes depending also on time [25].

In order to perform numerical simulations of the dynamical model (1) with *β* = *β*(*w*), *γ* = *γ*(*w*), *ϵ* = *ϵ*(*w*) and *ρ* = *ρ*(*w*), and estimate various moments of the solution, *N*(*t*; *w*), *S*(*t*; *w*), and *O*(*t*; *w*), we apply the Generalized Polynomial Chaos approach [26, 27].

In this context, polynomial chaoses can be arranged in a sequence Φ_*i*_((*w*)), such that the expansion of the random transmission parameters and stochastic processes appearing in the extended mathematical model (model (1) with random transmission parameters) takes the following form:

(3) $$\begin{array}{c} N\left(t;w\right)=\sum_{i=0}^{\infty }N_{i}\left(t\right)\Phi_{i}\left(\left(w\right)\right),\\ S\left(t;w\right)=\sum_{i=0}^{\infty }S_{i}\left(t\right)\Phi_{i}\left(\left(w\right)\right),\\ O\left(t;w\right)=\sum_{i=0}^{\infty }O_{i}\left(t\right)\Phi_{i}\left(\left(w\right)\right),\\ \beta \left(w\right)=\sum_{i=0}^{\infty }\beta_{i}\Phi_{i}\left(\left(w\right)\right),\;\;\gamma \left(w\right)=\sum_{i=0}^{\infty }\gamma_{i}\Phi_{i}\left(\left(w\right)\right),\\ \epsilon \left(w\right)=\sum_{i=0}^{\infty }\epsilon_{i}\Phi_{i}\left(\left(w\right)\right),\;\;\rho \left(w\right)=\sum_{i=0}^{\infty }\rho_{i}\Phi_{i}\left(\left(w\right)\right), \end{array}$$

where the Φ_*i*_ are properly chosen polynomial basis functions of some components of the random variable vector, and the number of variables in represents the dimension of the chaos (i.e., the number of input random parameters considered).

In this paper, since the probability distributions of the transmission parameters are uniform see Table 2, we have taken the expansions in terms of Legendre polynomials and = (*w*) as a vector with four components where each component is a random uniform variable with variation range in [−1,1]. Taking into account the orthogonality of the basis functions together with truncation of the polynomial chaos series to a finite number of terms will lead to an auxiliary system of ordinary differential equations governing the time evolution of the chaos coefficients of the solutions of the obesity model with random transmission parameters. In this paper, we will use a polynomial chaos method of order two based on Legendre polynomials (this means we will use Legendre polynomials up to degree two), and the chaos dimension is four (we consider four input random parameters: *β*, *γ*, *ϵ*, and *ρ*). Since there are fifteen Legendre polynomials of degree less or equal to two using a selection of the four variables of (there is one polynomial of degree zero, four of degree one, each one for each *ξ*_*i*_, four of degree two in one variable, each one for each (*ξ*_*i*_, *ξ*_*i*_), and six of degree two in two variables, each one for each (*ξ*_*i*_, *ξ*_*j*_), *i* ≠ *j*), the number of terms of the polynomial chaos expansion (truncation) of the unknown stochastic processes is equal to fifteen. In general this number is (*n* + *p*)!/(*n*!*p*!) where *p* is the maximum degree of the polynomials used, and *n* is the number of random parameters (*p* = 2 and *n* = 4 in this work). This number grows very fast with increasing *n* and *p*, which is one reason to choose the order of the chaos to be two. A more important reason is that in [27] a comparison was done for some epidemic models of the effect of the order on the solutions. There it was shown that while order one is not accurate, chaos or order two and three produce very similar results. A good reference to contextualize these assumptions considered related to the order of the polynomial chaos expansion is [26].

For *N*(*t*; *w*), for example, the chaos expansion will take the following form:

(4) N(t;w)=N0(t)+∑i=14Ni(t)Φ1(ξi(w))+∑i=14 ∑j=1iNij(t)Φ2(ξi(w),ξj(w)).

The first coefficient in the expression, *N*_0_(*t*), represents the first-order moment of the output stochastic process; *N*(*t*; *w*) and Φ_1_, Φ_2_ are Legendre polynomials in terms of a selection of the components of the vector. To be precise,

(5) $$\begin{array}{c} \Phi_{1}\left(\xi_{i}\left(w\right)\right)=\xi_{i}\left(w\right),\\ \Phi_{2}\left(\xi_{i}\left(w\right),\xi_{i}\left(w\right)\right)=\frac{3}{2}\xi_{i}{\left(w\right)}^{2}-\frac{1}{2},\\ \Phi_{2}\left(\xi_{i}\left(w\right),\xi_{j}\left(w\right)\right)=\xi_{i}\left(w\right)\xi_{j}\left(w\right). \end{array}$$

A proper description of the random transmission parameters in terms of the independent chaos variables *ξ*_1_(*w*), *ξ*_2_(*w*), *ξ*_3_(*w*), and *ξ*_4_(*w*) must take into account all the possible correlations between these parameters. Since we assume the four transmission parameters are independent random variables, each of them can be expanded as a functional of only one variable of *ξ*_1_(*w*), *ξ*_2_(*w*), *ξ*_3_(*w*), *ξ*_4_(*w*). Thus, its expansion to only order two is as follows.

(6) β(w)=∑i=0∞βiΦi((w))=β0+β1Φ1(ξ1(w))+β2Φ2(ξ1(w),ξ1(w)).γ(w)=∑i=0∞γiΦi((w))=γ0+γ1Φ1(ξ2(w))+γ2Φ2(ξ2(w),ξ2(w)).ϵ(w)=∑i=0∞ϵiΦi((w))=ϵ0+ϵ1Φ1(ξ3(w))+ϵ2Φ2(ξ3(w),ξ3(w)).ρ(w)=∑i=0∞ρiΦi((w))=ρ0+ρ1Φ1(ξ4(w))+ρ2Φ2(ξ4(w),ξ4(w)).

Note that *β*_0_, *γ*_0_, *ϵ*_0_, and *ρ*_0_ are the first-order moments of each transmission parameter.

We are now ready to develop the differential equations used in the numerical study. Considering the equations of the mathematical model (1) and introducing the polynomial chaos expansions, we obtain these equations.

Considering that we define the model in the restricted region {*N*(*t*) > 0, *S*(*t*) > 0, *O*(*t*) > 0, 0 < *N*(*t*) + *S*(*t*) + *O*(*t*) = 1} see [18], we can take into account that *N*(*t*) + *S*(*t*) + *O*(*t*) = 1, then it is only necessary to work with two of the equations of the system; for example, the second one and the third one and then determine *N*(*t*) from *N*(*t*) = 1 − *S*(*t*) − *O*(*t*). This option has also been considered in the polynomial chaos approach.

For notational convenience, we consider a one-to-one correspondence between the Legendre polynomials Φ_*q*_(·) (*q* = 1 and 2) and Ψ_*i*_(·). Then, *N*(*t*; *w*), for example, can be rewritten as *N*(*t*; *w*) = ∑_*i*=0_^*∞*^ *N*_*i*_(*t*)Ψ_*i*_. Now, taking into account this new notation and introducing the polynomial chaos expansions for *S*(*t*), *O*(*t*) and random transmission parameters in the last equation of (1), we obtain the following expression:

(7) ∑i=014ddtOi(t)Ψi=μO0+∑i=014γiSi(t)Ψi−μ∑i=014Oi(t)Ψi−∑j=014 ∑i=014ϵiOj(t)ΨiΨj.

To obtain a system of ordinary differential equations for the unknown coefficients with only one derivative of an unknown per equation, we use the orthogonality of the basis functions. In particular, taking the inner product of (7) with the basis functions Ψ_*L*_ (*L* = 0, 1,…, 14) results in

(8) 〈ΨL,ΨL〉ddtOL(t)  =〈μO0,ΨL〉+∑j=014 ∑i=014γiSj(t)〈ΨiΨj,ΨL〉   −μ∑i=014Oi(t)〈Ψi,ΨL〉   −∑j=014 ∑i=014ϵiOj(t)〈ΨiΨj,ΨL〉.

Note that 〈Ψ_*i*_, Ψ_*j*_〉 is defined as ∫_−1_^1^Ψ_*i*_Ψ_*j*_ *f*(*ξ*)*dξ*, and *f*(*ξ*) is the uniform probability density function.

For the second equation of system (1) the equivalent of the expression (8) is as follows:

(9) 〈ΨL,ΨL〉ddtSL(t)  =〈μS0,ΨL〉+∑j=014 ∑i=014βiOj(t)〈ΨiΨj,ΨL〉   −∑j=014 ∑i=014ρiSj(t)〈ΨiΨj,ΨL〉−μ∑i=014Si(t)〈Ψi,ΨL〉   −∑j=014 ∑i=014γiSj(t)〈ΨiΨj,ΨL〉   +∑j=014 ∑i=014ϵiOj(t)〈ΨiΨj,ΨL〉   +∑j=014 ∑i=014βiSj(t)〈ΨiΨj,ΨL〉   −2∑j=014 ‍∑i=014 ∑k=014βiSj(t)Ok(t)〈ΨiΨjΨk,ΨL〉   −∑j=014 ∑i=014 ∑k=014βiSj(t)Sk(t)〈ΨiΨjΨk,ΨL〉   −∑j=014 ∑i=014 ∑k=014βiOj(t)Ok(t)〈ΨiΨjΨk,ΨL〉.

Equations (8) and (9) are a nonlinear system of ordinary differential equations in the unknowns *O*_0_(*t*), *O*_1_(*t*),…, *O*_14_(*t*) and *S*_0_(*t*), *S*_1_(*t*),…, *S*_14_(*t*). This system (auxiliary system) will be solved numerically using an explicit Runge-Kutta method. Usually the quantities of interest are the first and second moments. The first moment, or expectation, is given, as we have mentioned by *S*_0_(*t*) and *O*_0_(*t*). *N*_0_(*t*) is calculated by 1 − *S*_0_(*t*) − *O*_0_(*t*). The calculation of the second moment (variance) will be given in the next section.

## 4. Sensitivity Analysis: Polynomial Chaos-Based Sobol' Indices

 A sensitivity analysis is also performed in order to quantify the output uncertainty due to the randomness in each of the transmission parameters. Polynomial chaos-based Sobol's indices are used. This method is based on the decomposition of the variance of the output as a sum of contributions of each input variable, or combinations thereof see [28, 29].

In order to compute the sensitivity indices based on the polynomial chaos expansions of the output stochastic processes it is necessary to consider the coefficients of these expansions, that is, *N*_0_(*t*), *N*_1_(*t*),…, *N*_14_(*t*), *S*_0_(*t*), *S*_1_(*t*),…, *S*_14_(*t*), and *O*_0_(*t*), *O*_1_(*t*),…, *O*_14_(*t*). Indeed, only elementary mathematical operations are needed to compute Sobol's indices from these expansion coefficients.

The idea behind the construction of polynomial chaos-based Sobol's indices is simple: once the polynomial chaos representation of the output stochastic process is available (the expansion coefficients are known, i.e., the solution of system (9) is known), the response expansion coefficients are simply gathered according to the dependency of each basis polynomial, square-summed and normalized. For example, the polynomial chaos-based Sobol's index which explains the influence of the parameter *β* on the stochastic process *O*(*t*; *w*), *SU*_*β*_, can be computed as follows:

(10) SUβ(t).=O12(t)Var⁡(Φ1(ξ1(w)))+O52(t)Var⁡(Φ2(ξ1(w),ξ1(w)))Var⁡(O(t)).

Note that Φ_1_(*ξ*_1_(*w*)), Φ_2_(*ξ*_1_(*w*), *ξ*_1_(*w*)) are the orthogonal polynomials involved in the definition of the parameter (in this case, parameter *β*) and *O*_1_^2^(*t*), *O*_5_^2^(*t*) are the coefficients of the chaos expansion of the process *O*(*t*; *w*) related to the orthogonal polynomials defined by *ξ*_1_(*w*): the random variable used to define *β*(*w*). Taking into account that *ξ*_1_(*w*) ~ *U*[−1,1], Var⁡(Φ_1_(*ξ*_1_(*w*))) and Var⁡(Φ_2_(*ξ*_1_(*w*), *ξ*_1_(*w*))) are computed. The value of the total variance, Var⁡(*O*(*t*)), can be calculate from the coefficients expansion obtained with the system of differential equations (8) and (9). In this case, for *O*(*t*) the variance is as follows:

(11) $$\begin{array}{c} \mathrm{Var}\left(O\left(t\right)\right)=\sum_{i=1}^{14}O_{i}^{2}\left(t\right)*\mathrm{Var}\left(\Psi_{i}\right). \end{array}$$

Note that numerator in (10) is a polynomial function depending on all random variables *ξ*_*i*_(*w*) related to random transmission parameters which we are analyzing, *β*, and only on them.

## 5. Results

### 5.1. Numerical Simulations

**Figure 1:**
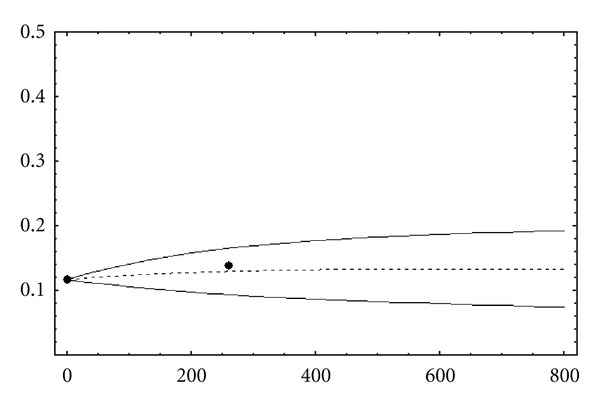
Prevalence prediction for obese subpopulation in the region of Valencia. Note that *t* = 0 correspond to year 2000 (the first week) and (•) are the obesity prevalence known by health surveys [19].

Figure 1 shows the results obtained for the obese sub-population using Legendre chaos. *O*_0_(*t*) is shown as a dotted line. In this and the next figures, we also plot the standard deviation interval, that is, plot the curves $O_{0}\left(t\right)\pm \sqrt{\mathrm{Var}(O\left(t\right))}$, $S_{0}\left(t\right)\pm \sqrt{\mathrm{Var}(S\left(t\right))}$, and $N_{0}\left(t\right)\pm \sqrt{\mathrm{Var}(N\left(t\right))}$, respectively. Note that for a fixed value of *t*, [$O_{0}\left(t\right)-\sqrt{\mathrm{Var}(O\left(t\right))}$, $O_{0}\left(t\right)+\sqrt{\mathrm{Var}(O\left(t\right))}$], for example, is a confidence interval in the sense known.

**Figure 2:**
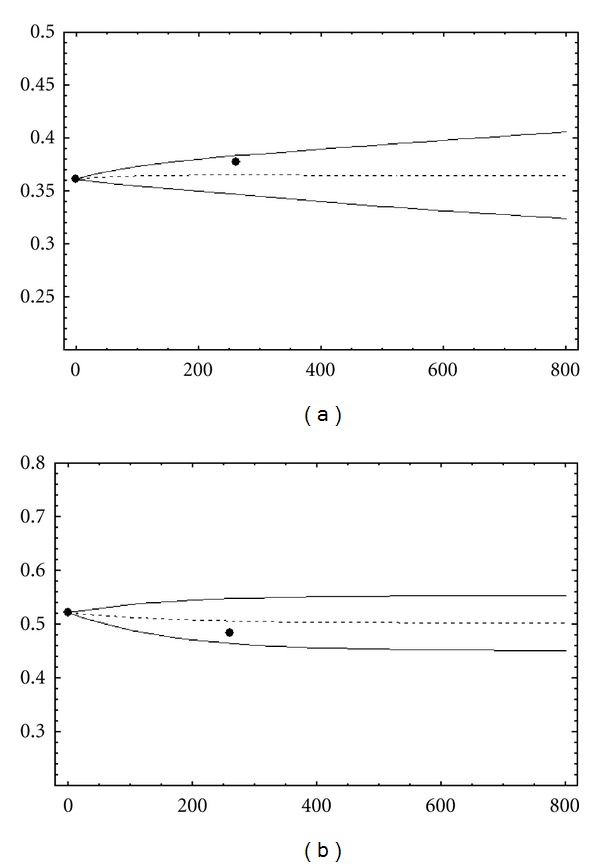
Prevalence prediction for overweight and normal-weight subpopulation in the region of Valencia. Note that *t* = 0 correspond to year 2000 (the first week) and (•) are the obesity prevalence known by health surveys [19]. (a): Overweight population. (b): Normal-weight population.

Figure 2 describes the overweight and normal weight prevalence for the next few years (until *t* = 800, year 2015). *S*_0_(*t*) and *N*_0_(*t*) are also shown as dotted lines. Some of the numerical values represented in Figures 1 and 2 are presented in Table 3.

We can observe that polynomial chaos approach quantifies the output uncertainty due to the randomness in the input parameters. The definition of the output confidence interval by second-order moment evaluation allows us to predict the epidemic evolution with more accuracy than in deterministic approach. As it is described in [18], we can note how the obesity epidemic in the region of Valencia, Spain, is increasing.  Table 4 shows the outcomes with fixed parameters and allows us to compare these outcomes with predictions performed by polynomial chaos approach (Table 3).

### 5.2. Sensitivity Analysis

**Figure 3:**
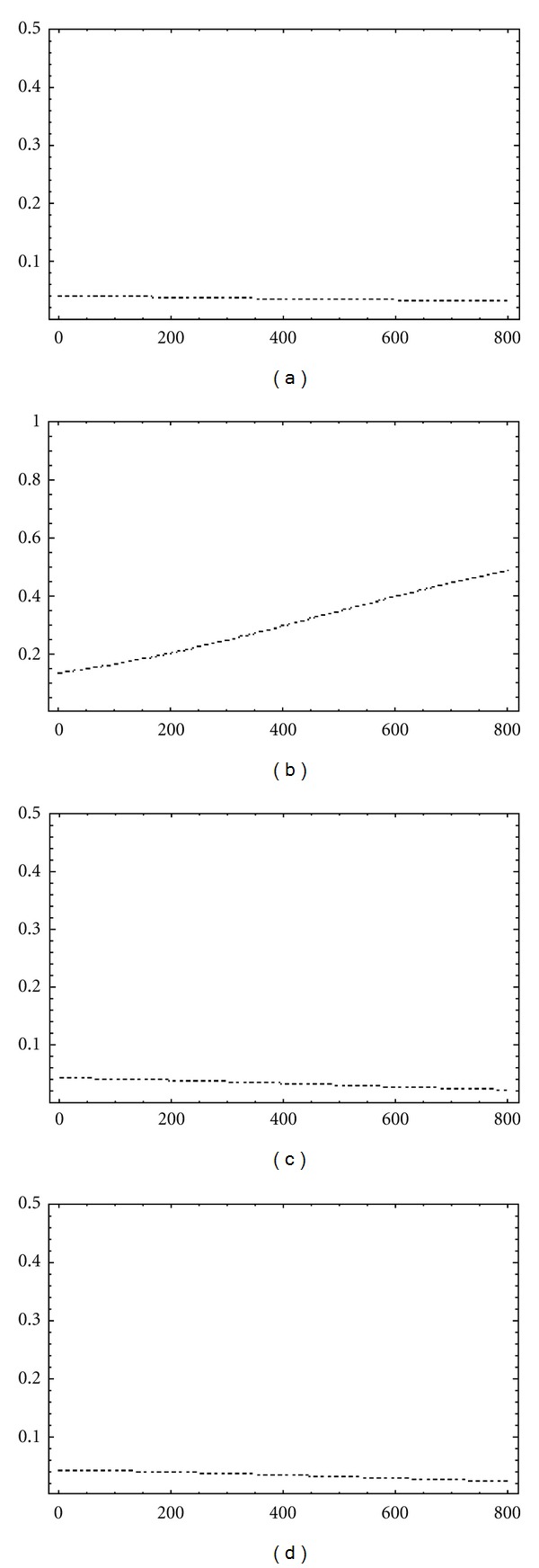
Influence of transmission parameters uncertainty on obesity epidemic prediction. Polynomial chaos-based Sobol indices. (a) *β* influence on obese population, *SU*_*β*_; (b) *γ* influence on obese population, *SU*_*γ*_; (c) *ϵ* influence on obese population, *SU*_*ϵ*_; (d) *ρ* influence on obese population, *SU*_*ρ*_.

Figure 3 shows the influence of parameters *β*, *γ*, *ϵ*, and *ρ*, respectively, in the prediction of obese population. Looking at *γ* contribution, it is clear that the epidemic evolution (i.e., variations of *O*(*t*)) depends on transit from overweight population to obese population. Therefore, if we assume that transmission parameters which lead to larger variations in the output (obesity prevalence in the next few years) define better options to control the obesity epidemic, we can conclude that prevention strategies related to overweight population can be an optimal policy to address the epidemic.

## 6. Conclusions

 In this paper, we have shown the possibilities of polynomial chaos related to epidemiological models. It is shown how polynomial chaos can be a useful tool to consider the effects of randomness on the evolution of the epidemics and to perform sensitivity analysis (by polynomial chaos-based Sobol's indices) in order to propose optimal policies to control epidemics.

As an example, we have studied an obesity model. As it is usual in social epidemic models, the transmission parameters involved in these types of mathematical model cannot be determined exactly, and it is necessary to introduce randomness. In this work, randomness in the transmission parameters is considered, and the resulting system of random coefficient differential equations has been solved approximately using the method of polynomial chaos.

We have shown how the application of polynomial chaos approach to an epidemiological model allows us to determine the epidemic evolution with more realism than in deterministic approach. Since in this case, it is possible to define a confidence interval to the epidemic evolution. Additionally, taking into account this approach, sensitivity analysis (an useful tool for policy makers and healthy planners) is easy to perform. Sensitivity indices based on polynomial chaos expansion may be computed with no additional cost.

To the best of our knowledge, this work is one of the first applications of polynomial chaos approach to epidemiological models based on ordinary differential equations although evidences detected make the method a good candidate to be employed in the study of epidemics.

## Figures and Tables

**Table 1:** Estimated parameters for the region of Valencia, Spain.

Parameter	Value
*β*	0.00085
*μ*	0.000469
*γ*	0.0003
*ϵ*	0.000004
*ρ*	0.000035
*N* ^0^	0.704
*S* ^0^	0.25
*O* ^0^	0.046

**Table 2:** Probability distributions of the transmission parameters.

Parameter	Value	Distribution
*β*	0.00085	Uniform (0, 0.0017)
*γ*	0.0003	Uniform (0, 0.0006)
*ϵ*	0.000004	Uniform (0, 0.000008)
*ρ*	0.000035	Uniform (0, 0.00007)

**Table 3:** Evolution of excess weight population for the next few years. Predictions are shown by standard deviation intervals. Additionally, mean values are presented.

Year	Overweight population	Obese population
2010	36.51%	13.16%
*t* = 520	[33.54%, 39.49%]	[8.13%, 18.18%]
2011	36.52%	13.18%
*t* = 572	[33.33%, 39.70%]	[7.96%, 18.39%]
2015	36.54%	13.21%
*t* = 780	[33.52%, 40.51%]	[7.36%, 19.05%]

**Table 4:** Evolution of excess weight population for the next few years using deterministic model (1) and parameter values shown in Table 1.

Year	Overweight population	Obese population
2010	37.86%	15.20%
2011	37.99%	15.52%
2015	38.14%	15.92%
